# Synthesizing controlled microstructures of porous media using generative adversarial networks and reinforcement learning

**DOI:** 10.1038/s41598-022-12845-7

**Published:** 2022-05-31

**Authors:** Phong C. H. Nguyen, Nikolaos N. Vlassis, Bahador Bahmani, WaiChing Sun, H. S. Udaykumar, Stephen S. Baek

**Affiliations:** 1 School of Data Science, University of Virginia, Charlottesville, VA 22903 USA; 2 Department of Civil Engineering and Engineering Mechanics, Columbia University, New York, NY 10027 USA; 3 Department of Mechanical Engineering, University of Iowa, Iowa City, IA 52242 USA; 4 Department of Mechanical and Aerospace Engineering, University of Virginia, Charlottesville, VA 22903 USA

**Keywords:** Design, synthesis and processing, Computational methods, Characterization and analytical techniques

## Abstract

For material modeling and discovery, synthetic microstructures play a critical role as digital twins. They provide stochastic samples upon which direct numerical simulations can be conducted to populate material databases. A large ensemble of simulation data on synthetic microstructures may provide supplemental data to inform and refine macroscopic material models, which might not be feasible from physical experiments alone. However, synthesizing realistic microstructures with realistic microstructural attributes is highly challenging. Thus, it is often oversimplified via rough approximations that may yield an inaccurate representation of the physical world. Here, we propose a novel deep learning method that can synthesize realistic three-dimensional microstructures with controlled structural properties using the combination of generative adversarial networks (GAN) and actor-critic (AC) reinforcement learning. The GAN-AC combination enables the generation of microstructures that not only resemble the appearances of real specimens but also yield user-defined physical quantities of interest (QoI). Our validation experiments confirm that the properties of synthetic microstructures generated by the GAN-AC framework are within a 5% error margin with respect to the target values. The scientific contribution of this paper resides in the novel design of the GAN-AC microstructure generator and the mathematical and algorithmic foundations therein. The proposed method will have a broad and substantive impact on the materials community by providing lenses for analyzing structure-property-performance linkages and for implementing the notion of ‘materials-by-design’.

## Introduction

Developing digital twins of multi-phase microstructures is increasingly important in materials modeling and characterization^1–3^. While physical experiments on real specimens may provide valuable ground-truth data for calibration and validation of material models, the process of acquiring and experimenting with physical specimens is often costly and laborious. In addition, the distribution of physical properties in nature-obtained specimens cannot be controlled easily, which forces materials scientists to rely on a numerous “cut-and-try” experiments. Furthermore, the discrepancies in microstructural heterogeneity^4^ and the limited reproducibility of experiments^5^ raise a fundamental question on the practicality of relying solely on experimental data to calibrate high-fidelity models^6^.

Instead, direct numerical simulations (DNS) can serve as a valuable complement to lab experiments. In DNS, computational experiments are conducted on microstructures inferred from microscopic imagery, such as micro computed tomography (μCT)^7^ or generated from 2D scanning electron microscopy (SEM)^8^. However, the route of using DNS to obtain structure-property-performance (SPP) relationships is still hindered by the constraints placed by image acquisition. Typically, only a limited number of images spanning a limited sample size is available from images. The images may also pertain to specific formulations, as parameterized by the size distributions of particles, defects such as voids and cracks, and other important morphological features. Hence, from a material design point of view, it would be desirable to cover a large and diverse collection of microstructures, spanning a broad regime of the configuration space. However, in reality, it is practically impossible to obtain such diverse samples, as there is no viable way to control the micro-morphology but to “cut-and-try”.

To overcome these limitations placed by available imaged datasets, there has been an increasing in the number of research activities involving stochastic microstructure reconstruction in the materials research community^9^. “Synthetic” microstructures can be generated from a large variety of approaches, including N-point correlation functions^10^, shape descriptors^11^, ellipsoid packing^12^, or Gaussian random fields^13,14^ with different sets of advantages and limitations. High-order N-point correlation functions may theoretically generate microstructures with consistent statistics attributes; nevertheless, the high computational costs of those approaches are intractable. Meanwhile, Gaussian autocorrelation is often limited to the case where the Gaussian stochastic process can be completely characterized by their second-order statistics; as a result, constructing microstructures as non-Gaussian fields may lead to more realistic reconstructed microstructures^15^. However, such a task is challenging in practice as it requires all the joint probability density functions to be determined or estimated. In brief, DNS performed on idealized synthetic images may not lead to inferred effective mechanical properties that are physically realistic and hence may only be valid for simple trend analyses^8^.

Machine learning (ML) based approaches have rapidly emerged as promising alternatives to overcome the limitations of traditional microstructure reconstruction methods^16–18^. In particular, convolutional neural networks (CNN) based deep learning (DL) approaches have been mostly investigated^19^. For example, Lubbers, Lookman, and Barros^20^, and Li and colleagues^21^ utilized high-dimensional features encoded in a pre-trained CNNs to develop numerical representations of microstructure morphology. They employed the VGG network^22^, a type of CNN that is popularly utilized in computer vision applications, had been pre-trained using a generic image classification dataset. The authors discovered that the neural activations in response to a microstructural image input yielded accurate and detailed characterization of the complex morphology of microstructures (i.e. “neural style”) and, thus, could serve as a *texture vector* corresponding to a given microstructure image. Furthermore, by employing the optimization-based texture synthesis formulation of Gatys et al.^23^, in which the objective of the optimization is to create a synthetic image yielding a texture vector similar to that of a real microstructure, they demonstrated that a CNN could be used to generate realistic synthetic microstructures. However, a significant drawback of such an approach is that the resultant synthetic microstructures can span only small variations in the texture space, as they are bounded to look similar to the reference image. Further, the user has no control over the material properties of generated microstructures, and the practical limitations of “cut-and-try” experiments still persist.

Recently, generative adversarial networks (GAN) based approaches for synthetic microstructure reconstruction have been drawing attention from the materials community. GAN is based on the competition between two neural network agents, namely *generator* and *discriminator*. In the context of synthetic microstructure reconstruction, the generator is a CNN that produces a synthetic microstructure image from a given stochastic noise, and the discriminator is another CNN that distinguishes if a microstructure image is synthetic or real. In principle, through the adversarial competition, the two randomly-initialized networks may eventually converge to a Nash equilibrium, in which synthetic microstructures generated by the generator are indistinguishable from real microstructures. Base on this idea, Chun et al.^24^ proposed a GAN architecture to parameterize the generator inputs so that the morphology of generated 2D microstructures could be controlled parametrically. The authors demonstrated that the GAN-based synthetic microstructures were more realistic and contained fewer artifacts compared to the outcomes of other CNN-based methods. However, many physical phenomena, including the microstructure deformation, the dynamic of fluid flow within a porous media, or the anisotropic material properties, cannot be adequately modeled by 2D microstructure images alone, rendering a need for the development of GAN to produce 3D synthetic microstructures^25^.

In the broader context of machine learning, there are 3D GAN solutions readily available to produce 3D models of common objects such as chairs, tables, and airplanes^26–28^. However, compared to these common objects, 3D material microstructures contain more complex geometric and topological structures not only globally but also locally. In contrast to the fact that the global configuration or ‘style’ of geometry is predominantly the most important result, for the design of materials, not only the global morphology of the microstructure but also the local patterns that comprise the global morphology become critical. Therefore, unlike the common object GANs, microstructure GANs must be able to parameterize both local and global geometric and topological configurations.

There are a few approaches that were proposed to overcome the above issues of microstructure 3D GAN. For instance, Mosser et al.^29^ stabilized the training of 3D GAN by introducing the Gaussian noise at the input of the discriminator and applying label-switching which can weaken the discriminator during the early stages. In another work, Hsu et al.^30^ tried to address the above issues by applying Wasserstein GAN (WGAN) in which the Wasserstein loss function^31^ was used to train the discriminator. The Wasserstein distance (WD) has good training properties as it is more sensible than other common distance function applied for GAN loss, including the Total Variation (TV) distance, the Kullback-Leibler (KL) divergence, and Jensen-Shannon (JS) divergence^31^. Therefore, WD allows GAN to learn the probability distribution of complex shapes more efficiently. Moreover, the training of WGAN is also more stable as even at training completion of the discriminator, loss still can be provide to the generator; thus, the effort for balancing the generator and discriminator training is no longer required^31^. Kench and Cooper^25^ overcome the difficulty in training microstructure 3D GAN differently by employing a discriminator that takes 2D microstructures images sliced from 3D synthetic model as the input instead of the fully 3D ones. The method has shown successes in producing high-quality 3D synthetic microstructures of different types of isotropic materials.

Despite of few achievements, previous works on 3D GAN microstructures are short in several aspects. First, the output 3D microstructures are not scalable beyond their fixed size by designed architecture^30^. Few works has proposed the employment of fully convolution architectures to overcome the scalability problems^29,32^. However, as mentioned by Kench and Cooper^25^, during the training, the spatial dimension of the input latent vector is set to 1; therefore, there is no kernel overlap at the first layer of the generator network. As a result, the quality of the output synthetic microstructures is questioned due to distortion. The author proposed expanding the spatial dimension of input latent space during training, as similar to the approach by Chun et al.^24^, so as the correlation between latent variables at different spatial locations can be properly modeled. Nevertheless, due to the training strategy, the approach by Kench and Cooper is only applicable for isotropic materials. Moreover, in most previous works, the quantitative linkages between the GAN parameters and physical properties are remained unknown. As a result, solving the inverse microstructure design problem, i.e. tuning of GAN morphology parameters that yields synthetic microstructures with realistic effective mechanical properties, is still a time-consuming task and difficult to accomplish manually. There are a few existing gradient-free optimization approaches to overcome such limitations of the GAN-based generative design methods^33^. However, the efficiency and the robustness of these gradient-free optimization approaches are often limited by the complexity of the design space^34,35^. Alternatively, conditional generative networks, which formulates the design constraints as additional inputs to the network, could also be considered as a viable option to address the limitations of the previous GAN approaches^36–38^. However, the critical drawback of conditional generative networks is that it does not guarantee the design constraints, as there is no feedback loop for ensuring the constrained properties.

This research extends our prior GAN-based approach^24^ for 3D microstructures along with the introduction of Actor-Critic (AC) reinforcement learning to tune the morphology parameters. We first make a substantive extension to the GAN method of Chun et al.^24^ to enable the generation of realistic 3D microstructures with arbitrary size and introduce several solutions to overcome the instability in the training of 3D GAN. Consequently, we augment the GAN model using the AC reinforcement learning model to create a new capacity to produce microstructures with desired target properties. The proposed method is validated on μCT scans of Bentheim sandstone, upon which we demonstrate that the GAN-AC framework is capable of generating visually and physically realistic microstructures. In addition, we also compare our GAN-AC framework with the Bayesian optimization (BO) to demonstrate its practical benefits over other conventional gradient-free design optimization approaches.

## Methods

### Overview

**Figure 1:**
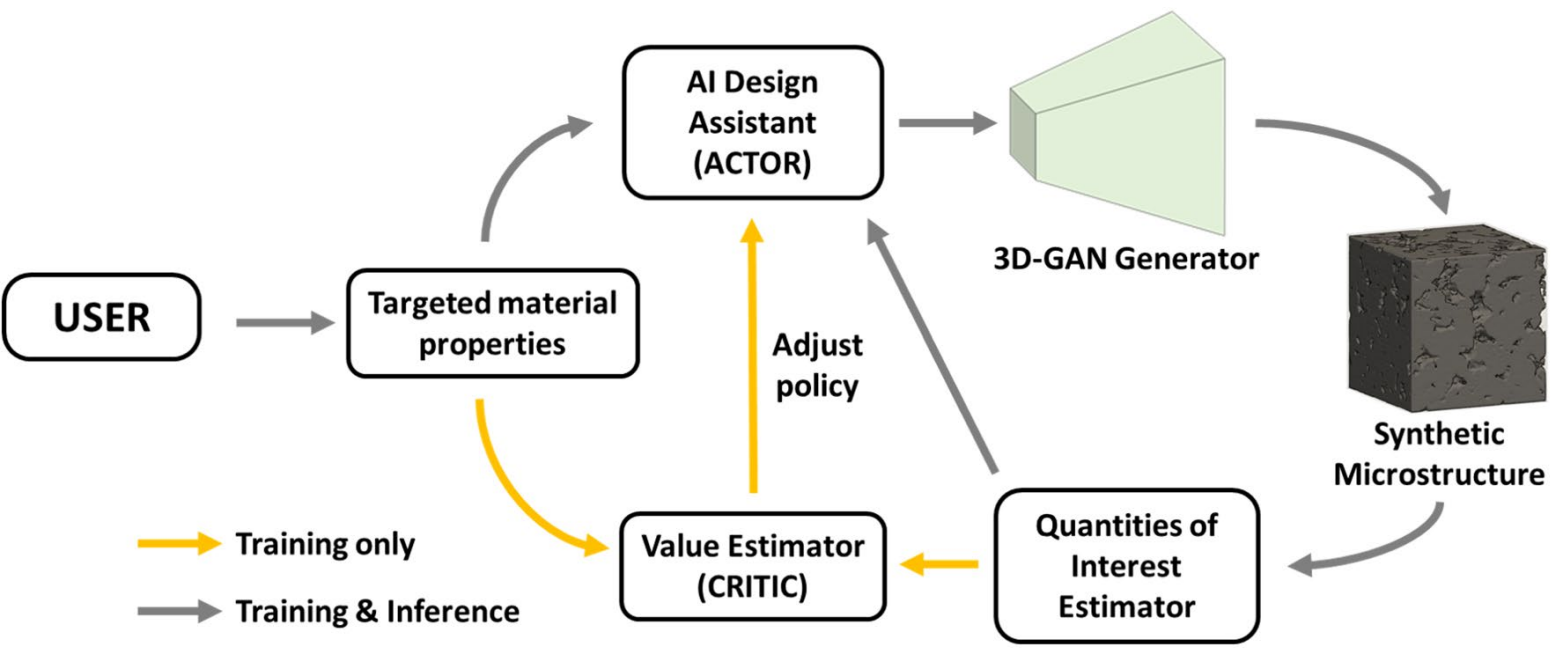
An overview of the proposed GAN-AC-based method. Initially, targeted QoI are provided by users. Consequently, the AI design assistant (the actor) starts to interact with the design environment by tuning input morphology parameters of the 3D-GAN generator for synthetic microstructure reconstruction. The properties of synthetic microstructures are estimated by DNS and sent to the value estimator (the critic) along with the targeted one to evaluate how good the design decision made the actor is. Based on the evaluation result, the actor’s policy will be refined to make better design decisions in the subsequent iteration. The training of the GAN-AC framework is completed with two steps: (1) train the 3D-GAN generator and (2) train the AC model. After training, the actor is retained for inference, while the critic is discarded.

As illustrated in Fig. 1, the goal of the proposed method is to generate a microstructure that yields targeted physical properties provided by the user. In our design framework, an AI design assistant, i.e. the *actor network*, tunes the input parameters for the *3D GAN*, in an attempt to achieve targeted physical properties. Consequently, the 3D GAN generates a 3D synthetic microstructure accordingly and OpenPNM, an open-source package for pore-network modeling^39^, performs analysis and evaluates the physical quantities of interest (QoI) of the synthetic 3D microstructure. The computed QoI are combined with targeted ones to be utilized by the *critic network* to evaluate how successful the actor’s action was in conforming to the targeted design goal. Finally, the *actor network* modifies its behavior, i.e. parameter tuning policy, based on the critic’s feedback, and the iteration continues until the actor becomes capable of generating microstructures with physical properties close to the target.

Training of the proposed algorithm is accomplished in two stages. In the first stage, the 3D-GAN is trained on samples of real microstructure images. As will be discussed later, the *generator network* learns to generate synthetic microstructure images and the *discriminator network* learns to distinguish real microstructure images from synthetic microstructure images. After a number of iterations, when these networks reach to the stage when the *discriminator network* is no longer able to distinguish between synthetic and real microstructure, the training process is complete. Consequently, the *discriminator network* is discarded while the *generator network* is retained to be used for synthetic microstructure reconstruction.

In the second training stage, the AC model is trained to solve the inverse microstructure design problem. Over a number of training episodes, the actor learns and refines its policy to generate morphology parameters for the 3D-GAN generator based on given targeted properties, while the critic learns to assess the quality of synthetic microstructures more accurately. Once trained, the critic network is discarded at the time of deployment.

### Synthetic microstructure generation using GAN

**Figure 2:**
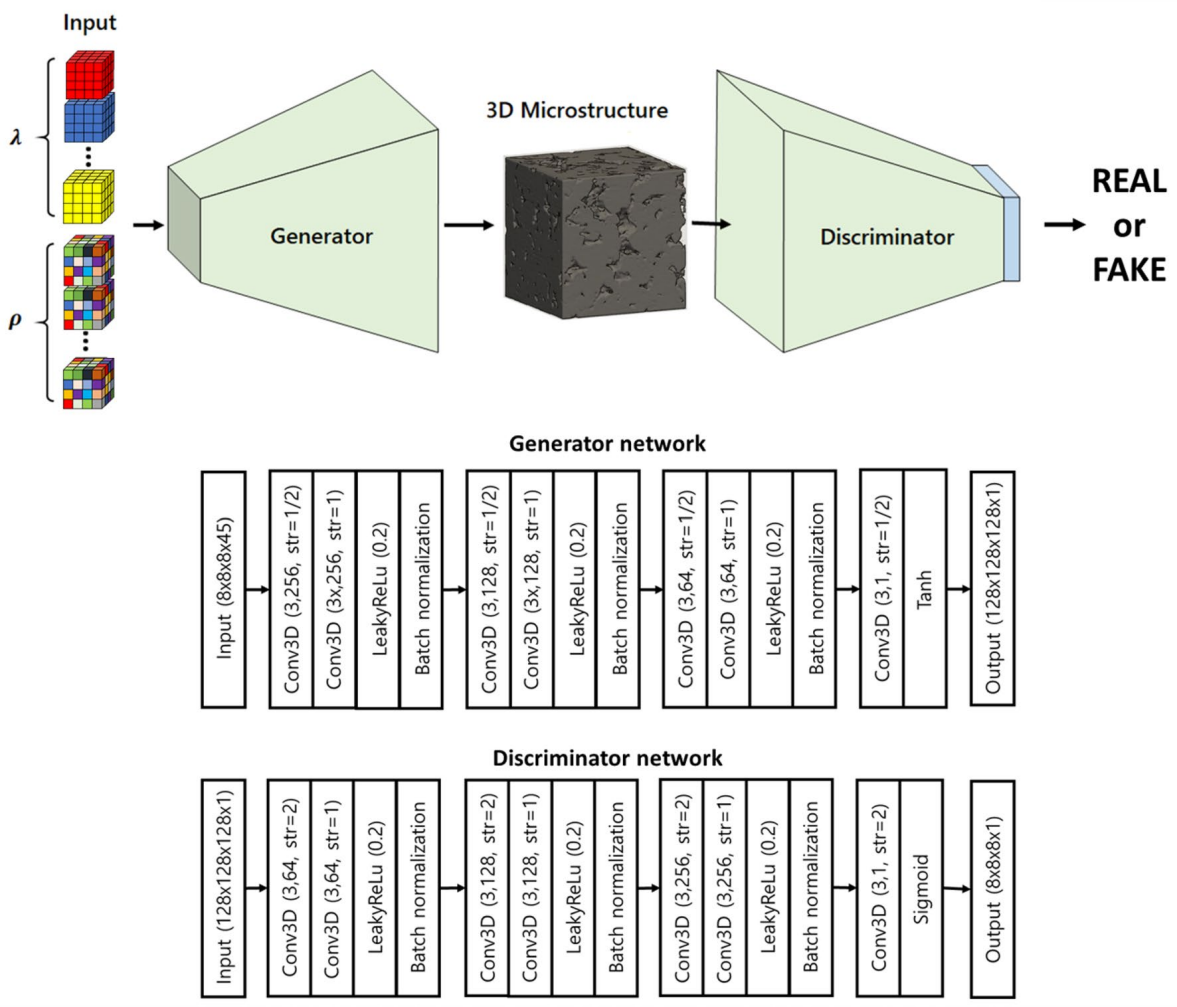
An overview of the 3D GAN architecture.

GAN has demonstrated abilities in generating more realistic microstructures compared to other CNN-based approaches^24^. Generally, GAN is trained via an adversarial competition (minimax game) of two neural networks, namely the *generator* ${\mathcal {G}}$ and the *discriminator* ${\mathcal {D}}$. During the training process, the generator ${\mathcal {G}}$ learns to generate realistic images to deceive the discriminator and the discriminator ${\mathcal {D}}$ attempts to distinguish real images from generated images. The training objective, i.e. the loss function, can be written as follows:

1 $$\begin{aligned} \min _{\mathcal {G}} \max _{\mathcal {D}} V({\mathcal {D}} , {\mathcal {G}} ) = {\mathbb {E}}_{x\sim p_{\text {data}}(x)} \left[ \log {\mathcal {D}} (x) \right] + {\mathbb {E}}_{z\sim p_z(z)} \left[ \log \left( 1-{\mathcal {D}} \left( {\mathcal {G}} (z)\right) \right) \right] , \end{aligned}$$

Here, $P_\text {data}$ denotes the distribution of the real images and *z* is the input parameter given to the generator. In principle, ${\mathcal {G}}$ and ${\mathcal {D}}$ should converge to a Nash equilibrium, in which the discriminator ${\mathcal {D}}$ is no longer capable of distinguishing real images from generated images. However, in practice, termination criteria are empirically determined for different problems.

Chun et al.^24^ advanced the above idea of GAN into a spatially parameterized GAN architecture for generating 2D synthetic microstructure images. Despite of their successes with 2D microstructure images, it is not straightforward to extend the architecture to 3D microstructures, mainly due to the morphological complexity of 3D microstructures compared to 2D. First, the 3D convolution layers has a significantly larger number of weights (i.e. network parameters to train) than the 2D version, which consequently increases the dimensionality of the optimization problem as well as the computational burden. In addition, the heavier network forces the use of a smaller batch size, which results in a longer training time and unstable convergence due to inaccurate approximation of the gradients. Furthermore, because 3D microstructures are much more sophisticated, 3D GAN requires a deeper architecture (i.e. more layers) for an increased expressiveness, worsening the numerical complexity even further.

As a result, in the current work, we introduce adjustments to the GAN architecture by Chun et al.^24^, to address these issues when extending the method for 3D (Fig. 2). First, the number of convolutional blocks in both the generator, ${\mathcal {G}}$, and the discriminator, ${\mathcal {D}}$, is reduced from five to four to reduce the complexity of the architecture and thereby to enhance the computational efficiency. In addition, for the first three convolutional blocks, we added a stride-1 convolutional layer after each half-stride (stride-2 for the discriminator) convolution layer to enhance the expressiveness of the 3D-GAN. Moreover, batch normalization layers are added at the end of the first three building blocks in both the generator and the discriminator networks, beside the use of leaky ReLU layers to avoid vanishing/exploding gradients and enhance the stability of the training process. Finally, kernels of size $3 \times 3 \times 3$ are used for all convolutional layers. With the new proposed architecture, each block of the generator network scales the dimension of the incoming tensor by a factor of 2, resulting in $128 \times 128 \times 128$ voxels as the final output for a given $8 \times 8 \times 8$ input tensor. Meanwhile, the discriminator is a ‘mirror image’ of the generator and produces a $8 \times 8 \times 8$ output tensor from a given $128 \times 128 \times 128$ microstructure image.

**Figure 3:**
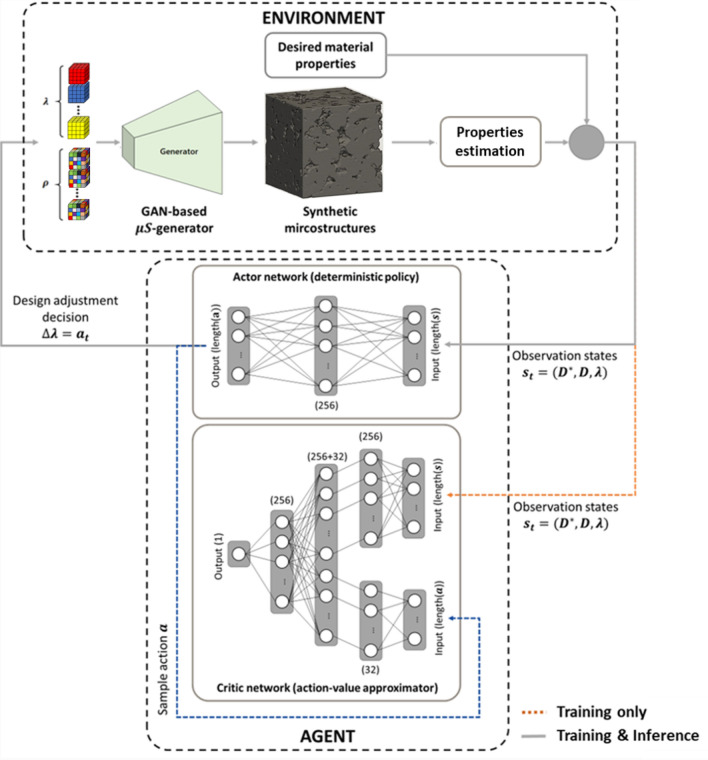
A schematic overview of the morphology control via an AC model. The design problem is formulated as a sequential process, adjusting global parameters at each step so that the synthetic microstructure transforms the morphology as desired. During the training process, *actor network* repeatedly gives design decisions based on its policy to interact with the *environment*. Physical properties of synthetic microstructures are computed in the *environment* and given as feedback to both *actor network* and *critic network*. The *critic network* then evaluates how successful the *actor network*’s action was in conforming the targeted properties. Finally, the *actor network* utilizes the evaluation from the *critic network* to adjust its policy in order to generate synthetic microstructures with physical properties closer to the targeted ones. Once the training is finished, the *critic network* is discarded and only the *actor network* is retained to perform the design task.

Similar to the original architecture^24^, our generator interfaces with two types of input parameters, namely the *global morphology* parameters, $\lambda \in {\mathbb {R}} ^{15}$, and the *local stochasticity* parameters, $\rho \in {\mathbb {R}} ^{30}$, defined at each location of an $8\times 8\times 8$ grid, resulting in a $8\times 8\times 8\times (15+30)$ input tensor. During the training time, $\lambda$, is kept constant across all $8\times 8\times 8$ grid locations of the input tensor, while $\rho$, randomly varies across different grid locations. Since the input $8\times 8\times 8$ grid is associated with $8\times 8\times 8$ overlapping regions in the output image (receptive fields), the microstructure morphology within each output region is controlled by the first 15 global morphology parameters, and the consequent 30, randomly varying local stochasticity parameters. This setting promotes the GAN to generate the same morphology or “style” across all output regions according to $\lambda$, while the local details could vary according to the randomly varying $\rho$.

In terms of training, ADAM^40^ optimizer with the learning rate of 0.0002 are used and all of data is normalized from [-1,1]. Furthermore, unlike the traditional GAN loss function, we evaluate Eq. (1) at each voxel and average them over the spatial dimension. Hence, the new loss function is defined as:

2 $$\begin{aligned} \begin{aligned} \min _{\mathcal {G}} \max _{\mathcal {D}} V({\mathcal {D}} , {\mathcal {G}} ) = \frac{1}{whd}\sum _{j=1}^{h} \sum _{i=1}^{w} \sum _{k=1}^{d} \left[ {\mathbb {E}}_{x\sim p_{\text {data}}(X)} \left\{ \log {\mathcal {D}} _{j,i,k}(x) \right\} + {\mathbb {E}}_{z\sim p_z(z)} \left\{ \log \left( 1-{\mathcal {D}} _{j,i,k}\left( {\mathcal {G}} (z)\right) \right) \right\} \right] , \end{aligned} \end{aligned}$$

where, ${\mathcal {D}} _{j,i,k}$ refers to discriminator’s prediction at voxel location (*j*, *i*, *k*). The application of the loss function as in Eq. (2) incentivizes the discriminator and the generator to scrutinize the details of microstructural patterns.

### Inverse synthetic microstructure reconstruction using actor-critic reinforcement learning

The inverse synthetic microstructure reconstruction task can be modeled as a reinforcement learning problem, whereby agents learn to take optimal actions to maximize long-term expected reward from given states of the environment. In the case of inverse synthetic microstructure reconstruction, the agents’ action is a series of adjustments in design parameters with the target of achieving desired QoI. Additionally, a given state of the environment should include information on the current design parameters, the current and the targeted QoI. Generally, the environment is modeled as a Markov decision process (MDP), denoted as ${\mathcal {M}} = \langle {\mathcal {S}},{\mathcal {A}},{\mathcal {P}},{\mathcal {R}}\rangle$. Here, ${\mathcal {S}}$ is the set of states $s_t$ that the agent can experience and ${\mathcal {A}}$ is the set of actions $a_t$ that the agent can take during its interaction with the environment. ${\mathcal {P}}: {\mathcal {S}} \times {\mathcal {A}} \rightarrow {\mathcal {S}}$ is a function which returns the probability over the state space, describing the likelihood of transition from the current state $s_t$ to the future state $s_{t+1}$ under an action $a_t$. Finally, ${\mathcal {R}}: {\mathcal {S}} \times {\mathcal {A}} \rightarrow {\mathbb {R}}$ is a reward function that defines rewards the agent can receive from the state $s_t$ transitioning under a particular action $a_t$. In the current microstructure reconstruction framework, the state $s_t$ consists of the current global morphology parameters of the generator, $\varvec{\lambda _t}$, the properties of the current synthetic microstructure, $\varvec{\mathrm {D}_t(\lambda _t)}$, and the targeted properties, $\varvec{\mathrm {D}^*}$. Also, the action $a_t$ determines the adjustment values $\varvec{\Delta \lambda _t}$ that are added to tune the material generator. The consequent state of the environment caused by action $a_t$ under state $s_t$ is $s_{t+1} = (\varvec{\lambda _{t}+\Delta \lambda _{t}, \mathrm {D}_{t+1}(\lambda _{t}+\Delta \lambda _{t}),\mathrm {D}^*})$. The reward function gives the total difference between targeted and synthetic normalized properties:

3 $$\begin{aligned} \begin{aligned} r_t(a_t,s_t) = \sum (\mathrm {D} - \mathrm {D}^*) \end{aligned} \end{aligned}$$

In Eq. (3), $\mathrm {D}$ and $\mathrm {D}^*$ are normalized QoI of synthetic and targeted microstructures with values varying between 0 and 1. Initially, relations between the states, the actor’s policy, and the critic are unknown. The goal of the reinforcement learning is to deduce those relations through repeated trial-and-error such that the optimal morphology parameters that yields the most realistic effective properties can be estimated.

The AC model we employ in this paper is comprised of two components. The ‘actor’ determines the action $a_t$ to interact with the environment based on the observed state $s_t$ via the policy function $\pi _\theta (a|s)$. The ‘critic,’ on the other hand, estimates the value function with given action (Q-value), $Q^\pi (s_t,a_t)$. Both actor and critic are modeled with deep neural networks. The objective is to learn an optimal policy which maximizes the cumulative reward, particularly in this case, minimizing the difference between synthetic QoI and the targeted ones, which can be written as:

4 $$\begin{aligned} \begin{aligned} J(\theta ) = \sum _{s \in {\mathcal {S}}} d^\pi (s) \sum _{a \in {\mathcal {A}}} \pi _\theta (a \vert s) Q^\pi (s, a) \end{aligned} \end{aligned}$$

where $d^\pi (s)$ is the stationary distribution of Markov chain for $\pi _\theta$. The problem of finding the optimal policy can be solved by utilizing policy gradient algorithm:

5 $$\begin{aligned} \begin{aligned} \nabla _\theta J(\theta )&\propto \sum _{s \in {\mathcal {S}}} d^\pi (s) \sum _{a \in {\mathcal {A}}} Q^\pi (s, a) \nabla _\theta \pi _\theta (a \vert s) \end{aligned} \end{aligned}$$

The AC algorithm is a well-known RL framework derived from a temporal different version of policy gradient where the strengths of both policy-based and value-based approaches are leveraged^41^. In the AC method, the two networks, $\pi _\theta (s)$ and $Q_w(a,s)$, are updated simultaneously during each training episode. While the actor adjusts parameters to find the optimal policy using Eq. (5), the critic adjust its parameters to minimize the difference between the function approximator $Q_w(a,s)$ and the true action-value function $Q^\pi (s, a)$^42^, such that the following is minimized.

6 $$\begin{aligned} \begin{aligned} {\mathbb {E}}_{s\sim d^\pi ,a\sim \pi _\theta }\left[ (Q_w(a,s) - Q^\pi (s, a))^2\right] \end{aligned} \end{aligned}$$

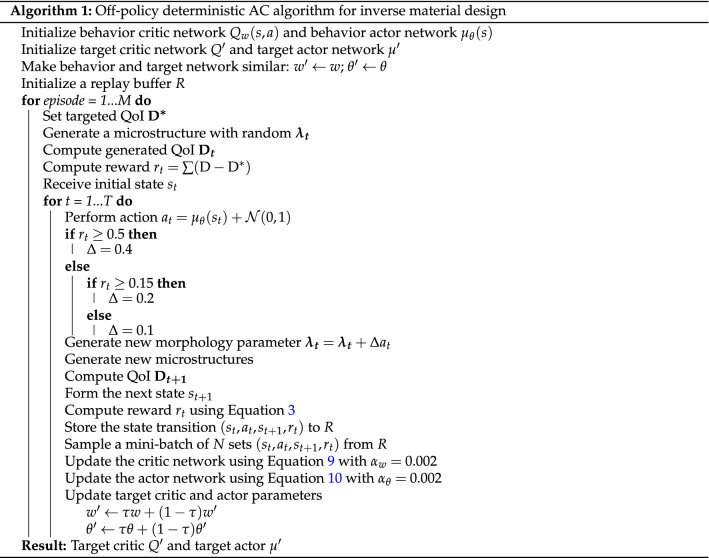

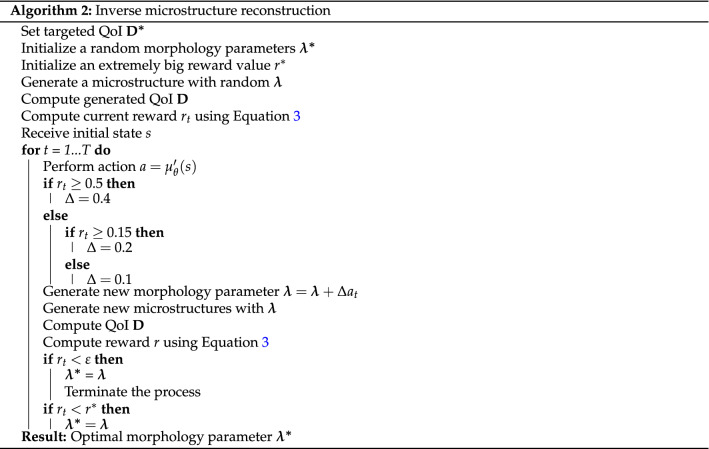


As studied in the work of Konda and Tsitsiklis^41^, the AC method tends to converge more smoothly and have better training performance for a system with a large state space.

In addition, since the synthesized microstructures are controlled with continuous morphology variables, a deterministic policy gradient (DPG) algorithm^42,43^ is applied. Particularly, in DPG, the actor decides the action to interact with the environment by a deterministic policy, such $a = \mu (s)$. As a result, the performance objective and the update scheme for both actor and critic are as follows:

7 $$J(\theta ) = \int _{\mathcal {S}} \rho ^\mu (s) Q(s, \mu _\theta (s)) ds$$

8 $$\delta _t= [r_t + \gamma Q_w(s_{t+1}, a_{t+1}) - Q_w(s_t, a_t)]^2$$

9 $$w_{t+1}= w_t + \alpha _w \delta _t \nabla _w Q_w(s_t, a_t)$$

10 $$\theta _{t+1}= \theta _t + \alpha _\theta \nabla _a Q_w(s_t, a_t) \nabla _\theta \mu _\theta (s) |_{a=\mu _\theta (s)}$$

Further, in this work, an off-policy AC method, which employs two different sets of actor-critic networks (behavior and target), is used. In off-policy AC, behavior networks take the role of exploration while the target ones take the role of learning to find the optimal morphology parameters. The application of off-policy AC brings two main advantages: (1) full trajectories are not required and the “experience replay” can be performed, and (2) the action sampling follows a behavior policy which is different from the target policy, providing better exploration. The training and inference processes are described clearly in Algorithms 1 and 2. In addition, the architecture of the proposed properties-driven morphology control utilizing AC algorithm is as in Fig. 3 and Gym^44^ is utilized for implementation. During training, the number of training episodes is set to 1000 with each episode containing 32 design iterations. Also, a total of 32,000 “experiences” can be saved in the replay buffer. A mini-batch size of 64 is used to update both actor and critic network using the ADAM optimizer^40^. Once the training is complete, the ‘target actor’ and ‘target critic’ are used for inference.Moreover, as similar to the training settings, the maximum of 32 design iterations are also set for the inference described in Algorithm 2.

**Figure 4:**
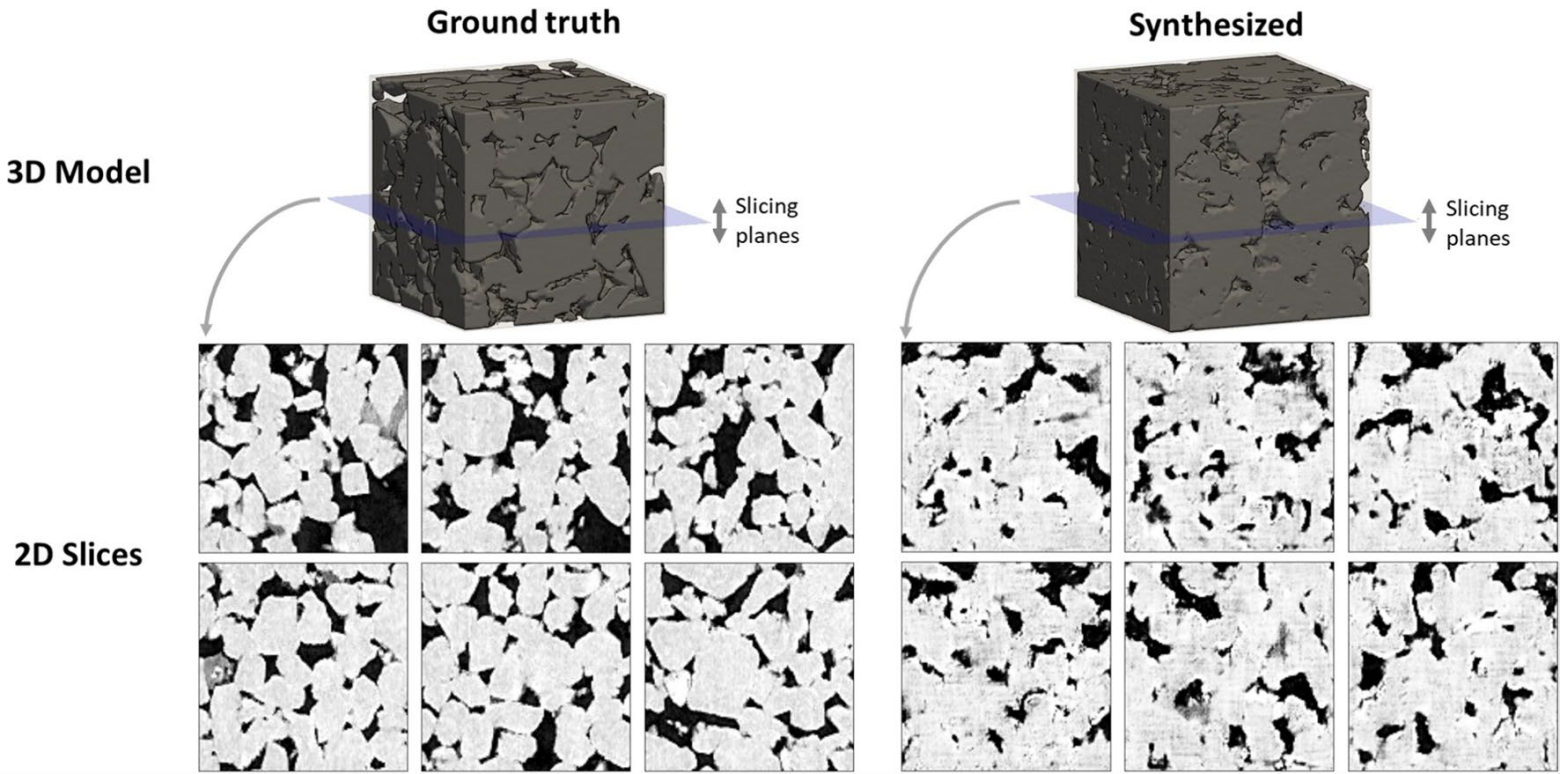
Comparison of 2D slices and 3D volume renders of ground truth microstructures and GAN-generated microstructures. In terms of the visual quality, the synthetic microstructures look realistic such that it is difficult to distinguish between the real and synthetic microstructures. Futher, the synthetic voids and crystals also look realistic and diverse.

## Results and discussion

**Figure 5:**
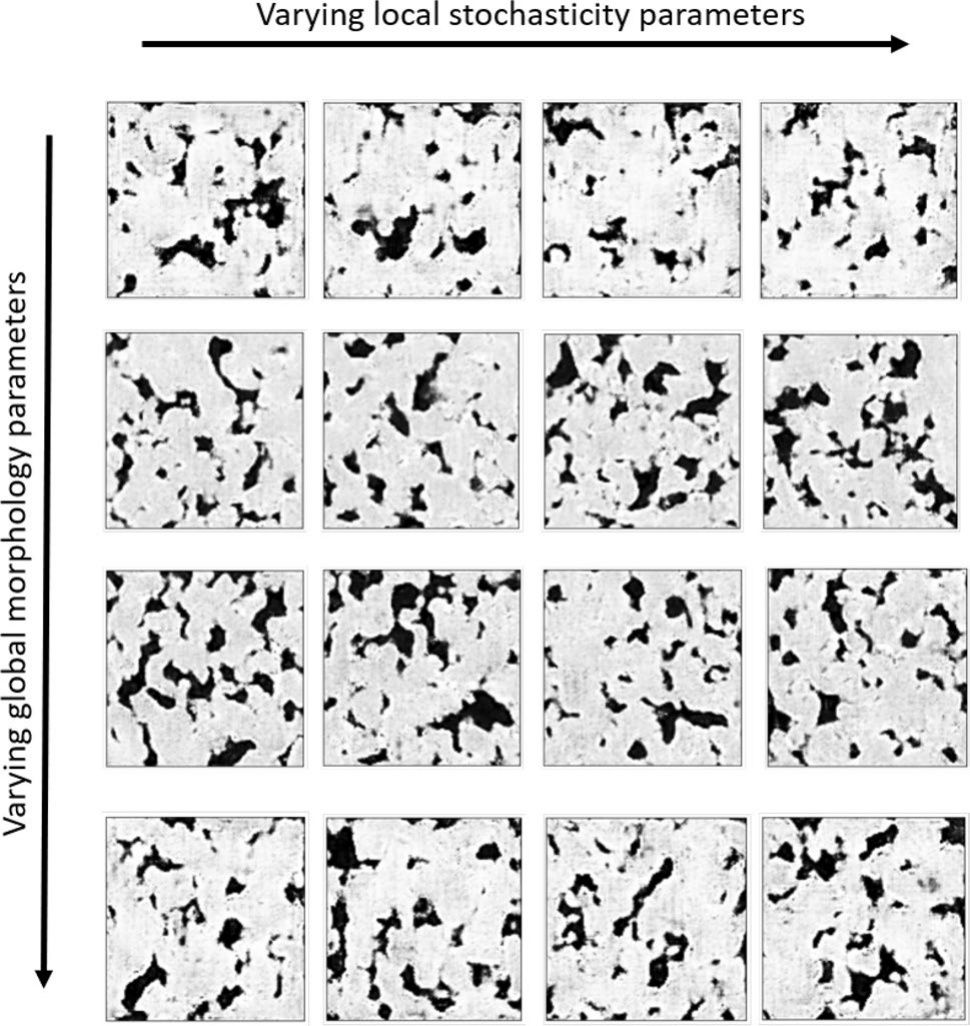
Effects of the local stochasticity parameter $\rho$. All images are 2D slices of synthetic microstructures taken by cutting planes at the same height position. Microstructures in the same row have the same morphology parameters but different local stochasticity parameters. As can be seen, their overall “style” is maintained and there are only subtle differences between microstructures slice images.

### Datasets

To test our microstructure reconstruction framework, we used a micro computed tomography (CT) scans data of Bentheim sandstones $(BM\_B1)$ provided by our colleague (Prof. Teng-fong Wong) as the training/validation data. The voxel size of the original data was $704 \times 678 \times 500$, from which $256 \times 256 \times 256$ sub-volumes were sampled at random positions and then coarsen into $128 \times 128 \times 128$. The resolution of the coarsened data is 8 micron per voxel and the size of representative elementary volume is $1024 \mu m \times 1024 \mu m \times 1024 \mu m$.

### Evaluation metrics

We use the following statistical, geometric, and topological attributes of the generated images and the real microstructures to measure whether the synthetic microstructures possess topological and physical properties consistent with the real specimen.

**Kullback–Leibler divergence (KLD)**^45^ is a statistical distance that can be used to measure how the distribution of syntehtic data $y \sim P_g$ differs from that of real data $x \sim P_r$:

11 $$\begin{aligned} KLD(P||Q) = {\mathbb {E}}_{{\varvec{x}}\sim P_r} \left[ log \frac{P_r(x)}{P_g(x)} \right] , \end{aligned}$$

where $P_r(x)$ and $P_g(x)$ are derived empirically using e.g., Kernel Density Estimation:

12 $$\begin{aligned} p(x) = \sum _{i=1}^{n}K({\varvec{x}},\varvec{x_i};h) \end{aligned}$$

where $K({\varvec{x}},\varvec{x'};h) \propto -\dfrac{||{\varvec{x}}-\varvec{x'}||^2}{2h^2}$ is the Gaussian kernel function and $x_i \in \{x_1, x_2, \ldots , x_N\}$ is a sample in the data set.

**Mean Maximum Discrepancy (MMD)**^46^ measures the disimillarity between two probability distributions using samples drawn independently from each distribution. Given a finite set of synthetic microstructures $x = \{\varvec{x_1},\varvec{x_2}, \ldots ,\varvec{x_m}\} \sim P_r$ and a finite set of real microstructures $y = \{\varvec{y_1},\varvec{y_2}, \ldots ,\varvec{y_n}\} \sim P_g$, the MMD of the two sets is defined as:

13 $$\begin{aligned} MMD = {\left[ {\mathbb {E}}_{{\varvec{x}}\sim P_r, {\varvec{y}}\sim P_g} \left[ K({\varvec{x}},\varvec{x'}) - 2K({\varvec{x}},{\varvec{y}}) + K({\varvec{y}},\varvec{y'}) \right] \right] }^{1/2}. \end{aligned}$$

**Earth Mover’s Distance (EMD)**^31^ is the minimum mass displacement to transform one distribution into the other, which is also known as the Wasserstein distance:

14 $$\begin{aligned} W(P_r,P_g) \propto \max _{f} {\mathbb {E}}_{{\varvec{x}}\sim P_r} \left[ f({\varvec{x}}) \right] - {\mathbb {E}}_{{\varvec{x}}\sim P_g} \left[ f({\varvec{x}}) \right] , \end{aligned}$$

where $f: {\mathbb {R}}^D \rightarrow {\mathbb {R}}$ is a Lipschitz continuous function, called the Wasserstein critic. In practice, *f* can be modeled as a neural network with clipped weights to have bounded derivatives, which is trained to derive high values at real samples and low values at generated samples, i.e. maximize:

15 $$\begin{aligned} {\hat{W}}({\varvec{x}}_{test}, {\varvec{x}}_g) = \frac{1}{N} \sum _{i=1}^{N} {\hat{f}} ({\varvec{x}}_{test} [i]) - \frac{1}{N} \sum _{j=1}^{N} {\hat{f}} ({\varvec{x}}_{g} [j]) \end{aligned}$$

**Pore space measurements** The pore space metrics measures the size and the tortuosity of the pore space and include the *total porosity* (ratio between the size of the void space and total volume) the *specific surface area* (the total area divided by the total volume). The total porosity is computed by the total number of void voxels in the cubic image divided by the size of the cube. The specific surface area is estimated by counting the total number of voxels that are at the interface between the void and solid constituent divided by the total volume of the cubic image.

**Effective permeability** The effective permeability of the GAN-generated micro-structure is the last and the most important metric that measures whether the GAN-generated micro-structures mimics the real micro structures. In brief, the effective permeability of a given porous medium to a fluid phase (oil, water, gas) is the ability of that phase to flow inside that medium given a hydraulic gradient^47^. In this work, the effective permeability is estimated with OpenPNM^39^.

**Topology measurements** The topology metrics measure is designed to measure the topological similarity of the pore connectivity graph generated from the GAN and those obtained from micro-CT imaging. To generate the connectivity graph, an open source software called PoreSpy^48^ is used to convert the binary images into weighted graphs and the properties of the weighted graphs are measured. The definition of these graph measures are listed below. They are calculated using the open-source software NetworkX^49^ for exploration and analysis of graph networks.

- **Degree assortativity**    The degree assortativity coefficient measures the similarity of the connections in a graph with respect to the node degree.
- **Graph transitivity**    The graph transitivity is the fraction of all possible triangles present in the graph over the number of triads. Possible triangles are identified by the number of triads—two edges with a shared vertex.
- **Graph density**    The density for undirected graphs is defined as $d=\frac{2 m}{n(n-1)}$, where *n* in the number of nodes and *m* is the number of edges of the graph.
- **Average clustering**    The average clustering coefficient of the graph is defined as $C=\frac{1}{n} \sum _{v \in G} c_{n}$, where *n* in the number of nodes. The clustering coefficient $c_n$ of node *n* is defined as $c_{n}=\frac{2 T(n)}{{\text {deg}}(n)({\text {deg}}(n)-1)}$, where *T*(*n*) is the number of triangles passing through node *n* and ${\text {deg}}(n)$ is the degree of node *n*.
- **Efficiency**    The efficiency of a pair of nodes is defined as the reciprocal of the shortest path distance between the nodes. The *local efficiency* of a node in the graph is the average *global efficiency* of the subgraph induced by the neighbours of the node. The *average local efficiency*, used in this work, is the average of the local efficiency calculated for every node in the graph.

### Evaluation of 3D-GAN synthetic microstructures

Figure 4 illustrates the comparison between the real and synthetic microstructures. By observing both 3D models and corresponding 2D slices, the synthetic microstructure is similar to the real microstructure as it is difficult to distinguish between them. Moreover, the shapes of voids and crystals of synthetic microstructures are also diverse and realistic, thus, showing that the proposed 3D-GAN-based material generator is capable of emulating the complex micro-geometry of natural materials. In addition, Fig.  5 shows the effects of the global morphology parameters and the local stochasticity parameters. As shown in the figure, the global morphology parameters result in the change in overall “style” of the synthetic microstructures, whereas the local stochasticity parameters create minor local variations in the microstructure morphology.

In addition, Table 1 quantitatively evaluates the quality of GAN-generated microstructures. For this comparison, we prepared two disjoint sets of real microstructures and a set of synthetic microstructures. Each of those three sets had 100 randomly selected samples. We computed the KLD, MMD, and EMD distances between the statistical distributions of real microstructures and synthetic microstructures. The statistical distance between the two sets of real microstructures were also computed to be used as the baseline for comparison. Results reported in Table 1 indicate that there is only negligible difference between real and synthetic microstructures, in terms of their statistical distributions.

**Table 1:** Evaluation of synthetic microstructures generated by GAN.

Metric	Synthetic–real	Real–real
Kullback–Leibler divergence (KLD)	0.0235	0.0197
Maximum mean discrepancy (MMD)	0.0190	0.0175
Earth mover’s distance (EMD)	0.0799	0.0899

**Figure 6:**
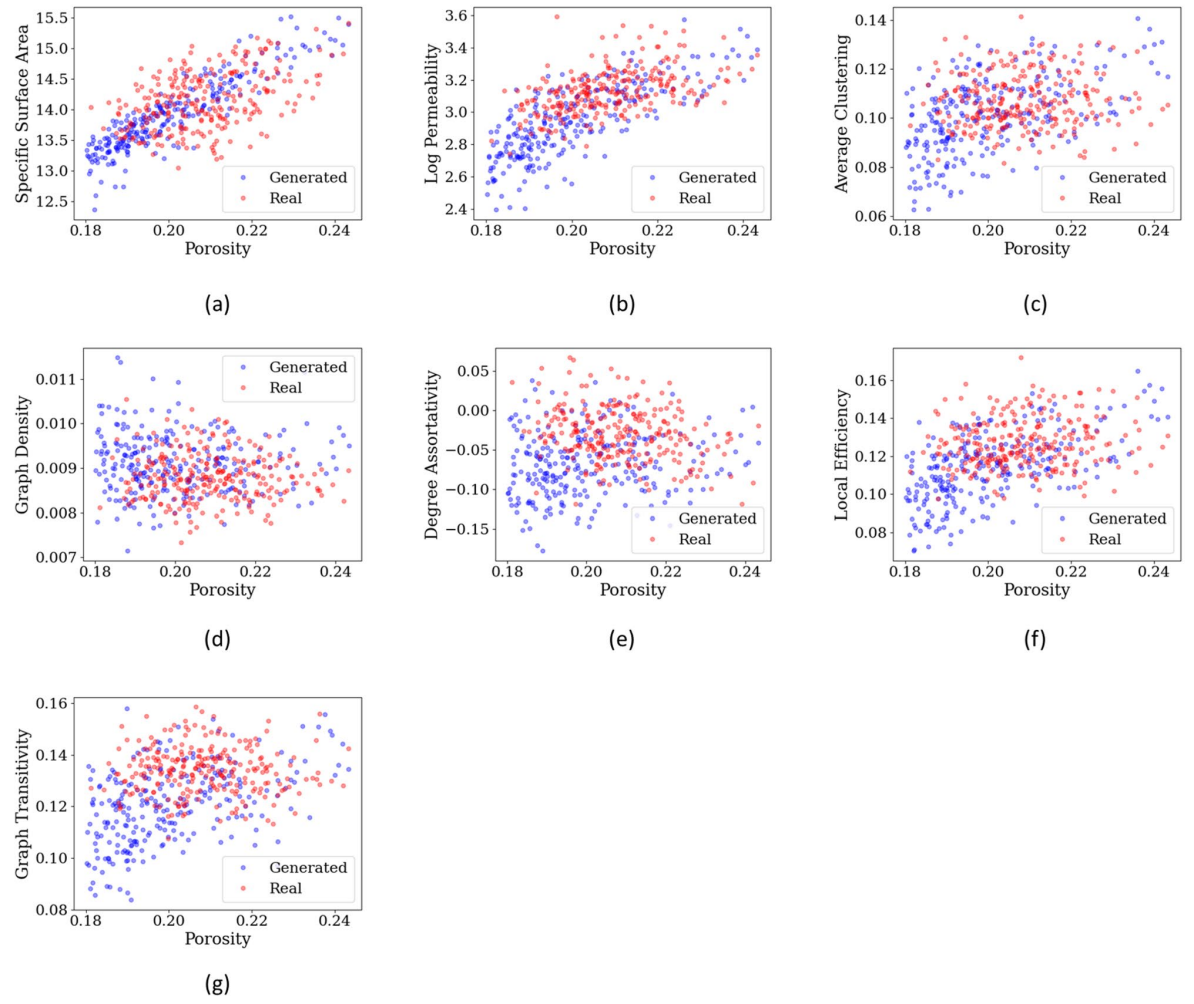
Quantitative evaluation of 3D synthetic microstructures. Pore space measurements, topology measurements, and permeability are plotted w.r.t porosity. (**a**) specific surface area, (**b**) log permeability (md), (**c**) average clustering, (**d**) graph density, (**e**) degree assortativity coefficient, (**f**) local efficiency, and (**g**) graph transitivity. As seen in the plots, there is an agreement between physical properties of ground truth microstructures and synthetic microstructures; thus, proving the validity of the proposed 3D-GAN microstructure generator.

Furthermore, we also validated GAN-generated microstructures using several physics-based metrics, including porosity, specific surface area, permeability, average clustering, graph density, degree assortativity coefficient, local efficiency, and graph transitivity. As reported in Fig. 6, physical properties of synthetic microstructures are, in general, in a good agreement with those of the real microstructure data and the distributions of the properties coincide with each other. Moreover, it is worth noting that the proposed 3D-GAN generator can generate synthesized microstructures with porosities that cover the range between 0.1 and 0.25 which is larger than that of the ground truth which only covers porosities between 0.18 to 0.25. This extrapolation capacity could be helpful on extending the material databases for characteristics provided that the micro-structural attributes of RVE outside the training data range remain sufficiently similar^6^.

### Evaluation of the GAN-AC framework for synthetic microstructure reconstruction

**Figure 7:**
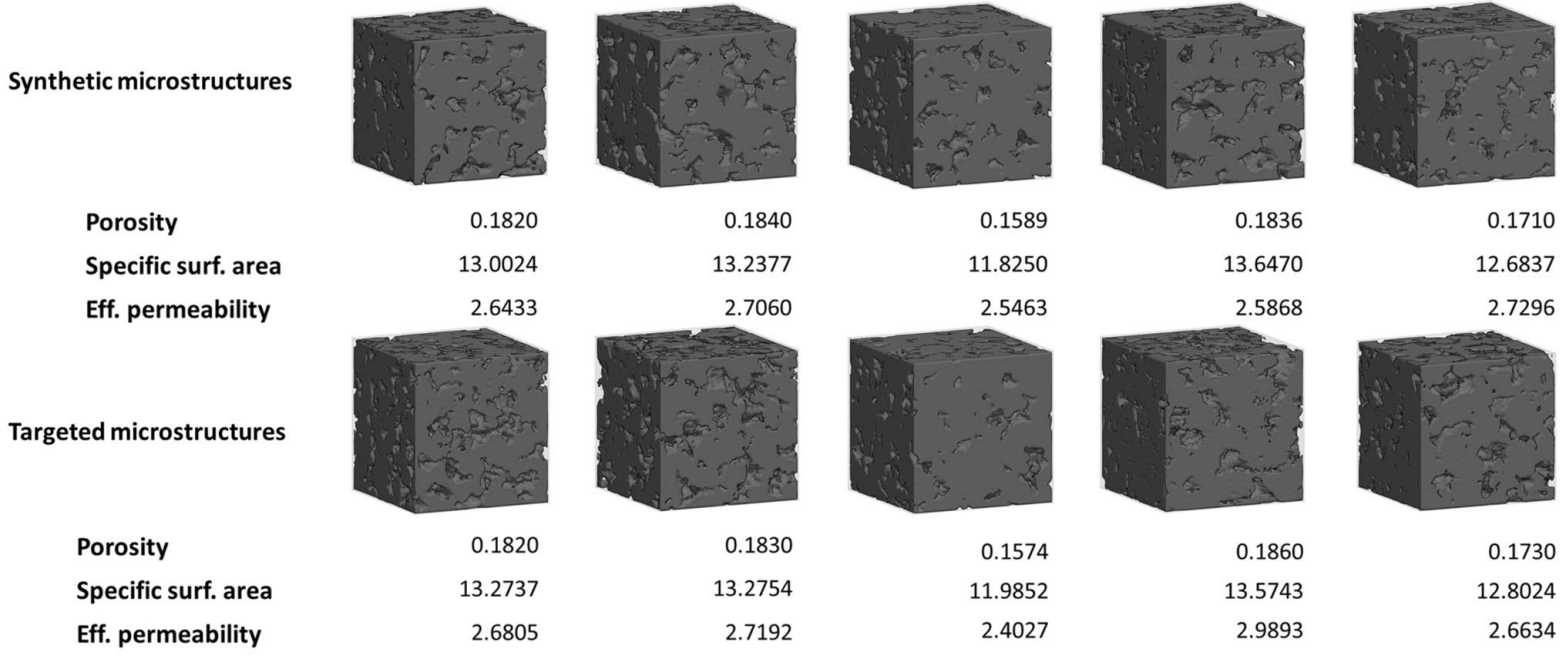
Visualization of synthetic microstructures generated by the proposed GAN-AC framework (top) and their corresponding targeted microstructures (bottom). The 3D voxelized data has been made available to public for inspection and third-party validation.

**Figure 8:**
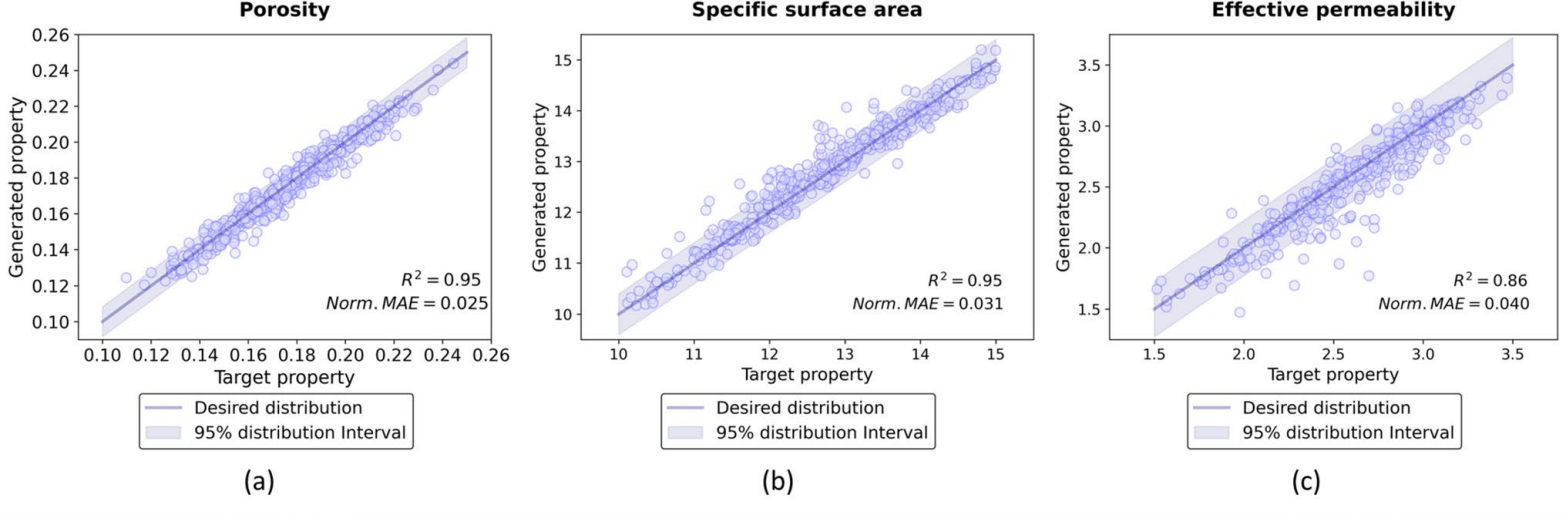
Comparison between targeted and generated properties. On the test conducted on 500 examples that were set aside during training, synthetic microstructures generated by the GAN-AC framework exhibited the targeted physical properties with extremely small difference, 2.54, 3.51, and $4.06\%$ for porosity, specific surface area, and effective permeability, respectively.

**Figure 9:**
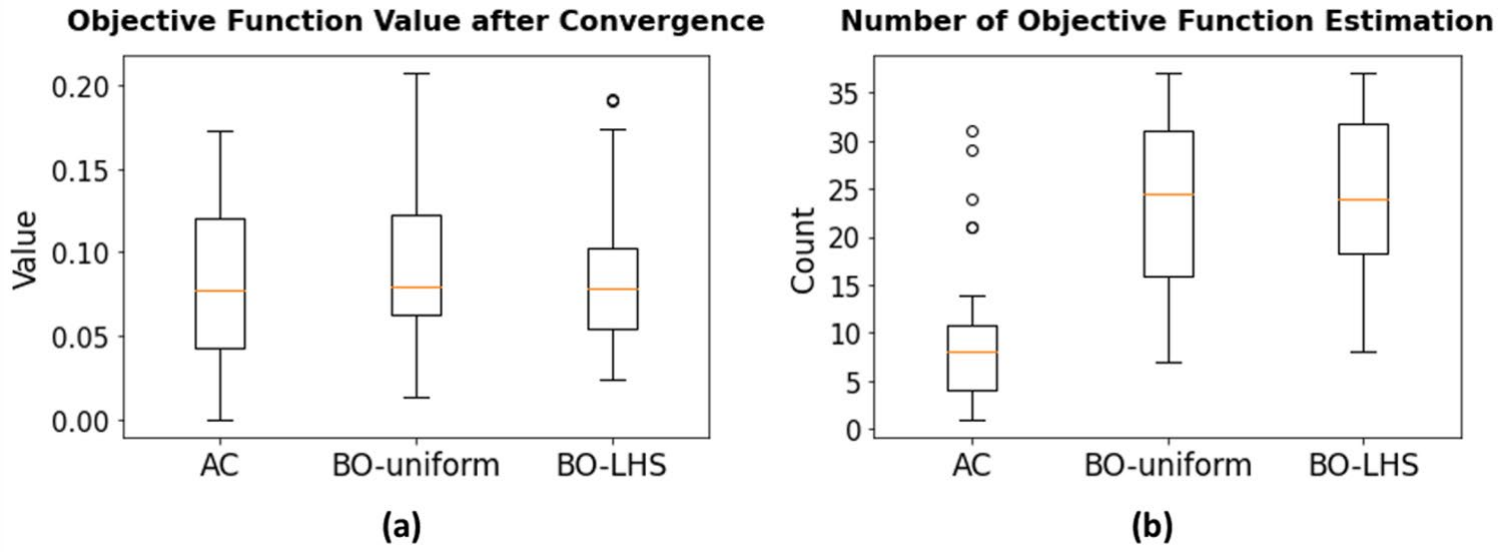
Performance comparison between our GAN-AC framework and a conventional optimization approach (Bayesian optimization; BO). (**a**) While the quality of the solution is on par compared to BO with Latin Hypercube sampling and marginally better than BO with random uniform sampling, (**b**) the AC method is significantly more efficient than BO with both initial sampling strategies (about three times) in terms of the number of objective function evaluation.

The validation of the GAN-AC framework is conducted via a microstructure reconstruction problem with targeted QoI including porosity, specific surface area, and effective permeability. The 3D-GAN model is utilized as the microstructure generator and an AC model, trained by using Algorithm 1, is attached to control the morphology parameters of the generator. The inverse design process follows Algorithm 2.

Figure 7 shows some examples of synthetic microstructures generated by the proposed GAN-AC framework with their physical properties. Additionally, the referenced microstructures with targeted physical properties were added for comparison. Generally, although synthetic microstructures presented in Fig. 7 are visually different with the targeted ones, their physical QoI are relatively similar. Moreover, Fig. 8 illustrates the quantiative evaluation of 500 synthetic microstructures by comparing the synthetic and targeted QoI. As illustrated, the synthetic microstructures can exhibit the properties of targeted ones with the average error of 2.54, 3.51, and $4.06\%$ for porosity, specific surface area, and effective permeability, respectively. These evaluation results demonstrate the validity of the GAN-AC framework in terms of inverse microstructure design. We have also made 10 synthetic 3D microstructures available via an open-access data repository, Mendeley data.

### Comparison with other design optimization approaches

Bayesian optimization (BO) is a well-known gradient-free optimization technique that is popularly applied for solving inverse design problems in material science^33,50^. Acknowledging its state of the art performance, we use BO as a reference of comparison to further investigate the performance of our proposed GAN-AC framework. For the implementation of BO with the GAN microstructure generator, we employ the following steps. First, an initial dataset is created by sampling within the 15-dimensional (global parameters) GAN latent space. Second, the QoIs of the initial microstructures are computed and the Gaussian process model is fit to the initial data. From the predicted uncertainty of the Gaussian process model (acquisition function), the next sampling point is determined. Finally, the QoIs of the new sample are estimated and added to the database for updating the Gaussian process model and the current solution. The same process is repeated until the convergence criteria is met or the algorithm reach the maximum number of iterations.

As similar to the AC method, BO also targets to minimize the objective function in Eq. (3) which measures the difference between desired and generated QoIs. We selected expected improvement (EI) as our acquisition function to balance between exploitation and exploration^50^. In addition, the maximum number of iterations is set to 32 to make its working condition similar to that of the GAN-AC model during inference. Finally, since the performance of BO is highly affected by intital sampling, we examined two different sampling strategies, including Latin Hypercube sampling (BO-LHS) and random uniform sampling (BO-uniform). In addition, the number of initial samples is set to 5 empirically, to assure the best quality of BO solution with the lowest number of objective function estimations.

Figure 9 shows the performance comparison between AC and BO. As can be observed from Fig. 9a, both the AC and BO exhibited a similar performance in terms of the converged minimum. In particular, while AC-computed minima were $0.0836 \pm 0.0479$, the BO-uniform-computed minima were $0.0934 \pm 0.0450$ and BO-LHS-computed minima were $0.0842 \pm 0.0407$, for 50 different optimization tasks with various target QoIs. In addition, despite of requiring 1000 training optimization instances, AC was significantly (about 3 times) faster in terms of the number of objective function evaluation, in which AC only required the average of 8.7 function evaluations whereas BO-uniform required 23.7 function evaluations and BO-LHS required 24.4 function evaluations on average.

In fact, it is widely accepted that reinforcement learning approaches in general are more efficient and scalable than conventional design optimization approaches (e.g., BO or genetic algorithm)^34,35,51^. The problem of generating microstructures is highly complex because the wide variation of grain/pore sizes, aspect ratios, orientations, and many other morphological attributes spans a vastly large design space. While traditional optimization methods are often limited by the complexity of the design problems^34^, the proposed GAN-AC framework can be significantly faster than traditional optimization algorithms during the inference time, even though the training could be computationally demanding and data-intensive. Therefore, for design optimization applications where the same type of material needs to be repetitively optimized for different design targets, reinforcement learning approaches provide a substantial advantage over traditional optimization algorithms^34,35^. In addition, reinforcement learning approaches are problem-aware, as opposed to the traditional optimization approaches that are problem agnostic. Reinforcement learning (RL) algorithms can learn the “landscape” of the design space, and they become better at searching for the nearest optimum as they accumulate more experiences, making it more generalized and is suitable for repetitive optimization tasks^52^. Also, it is worth to note that, the generalizability of the proposed GAN-AC method is only stopped at unseen optimization tasks where only targeted QoI values are different. For the design problems whereby different types of targeted QoI or different materials are required, BO is still more beneficial as RL requires a retraining of the model. Although methods such as transfer learning can reduce the effort of retraining RL algorithms, their effectiveness is still need to be further investigated.

## Conclusions

In this work, a novel method for inverse synthetic microstructure reconstruction utilizing 3D-GAN and off-policy deterministic AC reinforcement learning is proposed. Experimental studies on a dataset collected from X-ray CT scans of a Bentheim sandstone justified the validity of the proposed method. As reported, the synthetic microstructures are diverse and realistic in qualiative and quantiative comparison with the real microstructures. Further, the application of the AC model also enables the controllability on the physical properties of synthetic microstructures. The quantiative analysis of three given physical properties, including porosity, specific surface area, and effective permeability, results in a good agreement between synthetic and targeted physical QoI. The results demonstrate the capability of the proposed method in mimicking original microstructures both geometrically and physically.

For our future work, there are a few extensions to be made to make the GAN-AC framework more practical and usable. For example, a multi-agent AC model with multiple actors could be adopted to accelerate the explorations. Multi-actor models with decentralized policies and shared experience, for instance, may enable more effective exploration-exploitation than the current single-actor counterpart. Furthermore, we may consider capturing the alestoric and epistemic uncertainties of real microstructures. Uncertainty quantification is necessary in practice, as the process-structure-property relationships of real-world materials incorporates a great deal of stochasticity, arising from manufacturing, measurement, and modeling errors^53^. Such randomness could be addressed by solving a stochastic inverse problem whereby a stochastic solution, instead of a deterministic one, is derived. In addition, the manufacturability, or more broadly, the process-structure relationships were not included in the scope of this current work, which must be further explored as an immediate future work. Finally, physics-informed machine learning, in which the neural network architecture is designed to fulfill the relevant physical constraints (e.g. material symmetry, invariance properties), can also be applied to further assure the validity of the synthetic microstructures.

## Data Availability

The data set generated and analyzed during the current study are available in Mendeley Data (https://data.mendeley.com/datasets/tp9nynzc34/1).
